# Supplementary material for: Association between oral microbiome and seven types of cancers in East Asian population: a two-sample Mendelian randomization analysis
Source: Front Mol Biosci. 2023 Nov 21;10:1327893. doi: 10.3389/fmolb.2023.1327893 (PMC10702768; doi:10.3389/fmolb.2023.1327893)
Supplement: Supplementary file 1 [file DataSheet1.ZIP › Supplementary Materials/MR plots for tongue/tongue═╝/Pancreatic cancer/╥╚╧┘░⌐_╔╕╤í╜ß╣√/pheno.2291_to_pancreatic cancer_forest.pdf]

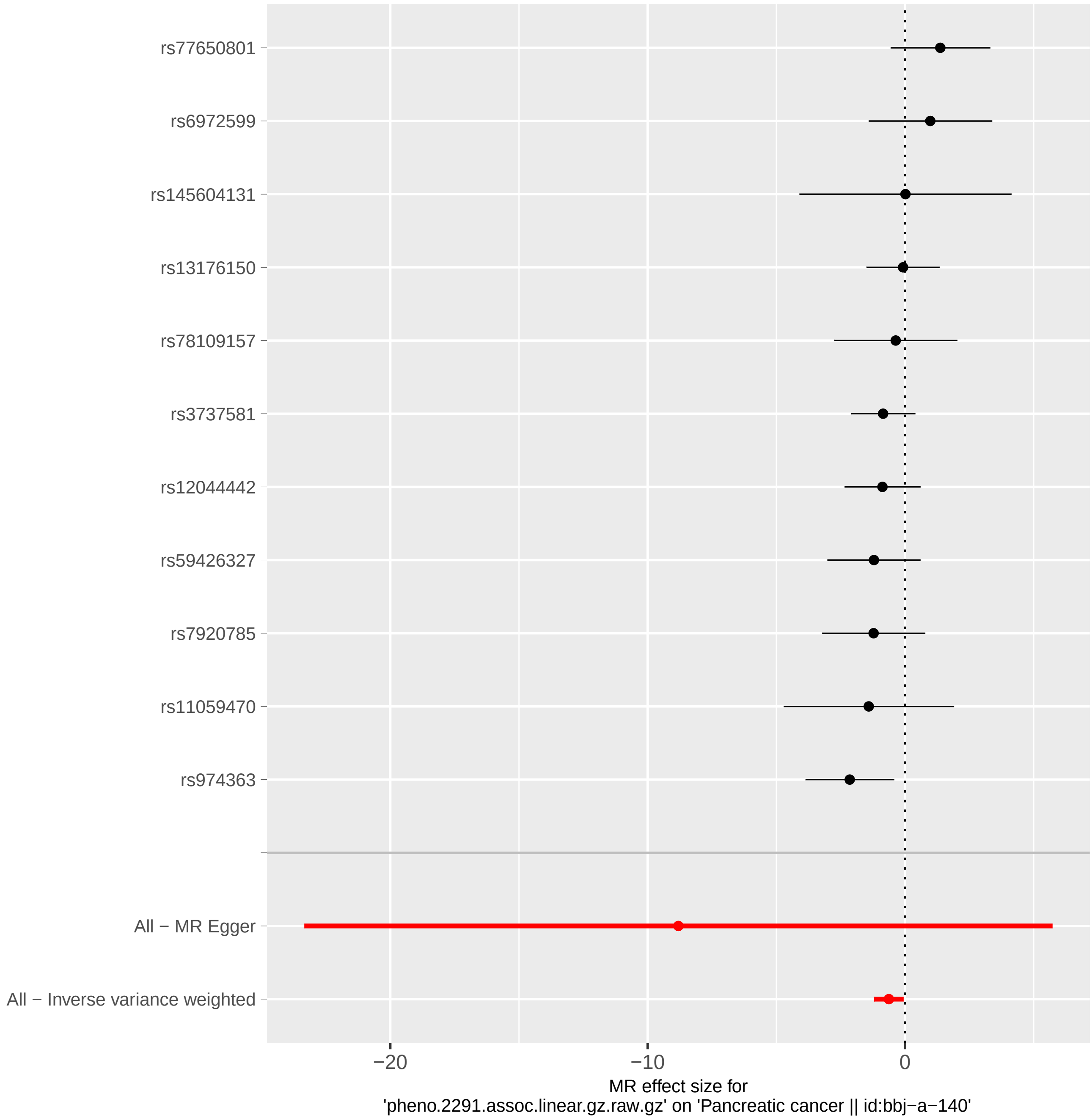

rs77650801
rs6972599
rs145604131
rs13176150
rs78109157
rs3737581
rs12044442
rs59426327
rs7920785
rs11059470
rs974363
All − MR Egger
All − Inverse variance weighted
−20
−10
0
MR effect size for
'pheno.2291.assoc.linear.gz.raw.gz' on 'Pancreatic cancer || id:bbj−a−140'
